# Inhibiting ATP6V0D2 Aggravates Liver Ischemia-Reperfusion Injury by Promoting NLRP3 Activation via Impairing Autophagic Flux Independent of Notch1/Hes1

**DOI:** 10.1155/2021/6670495

**Published:** 2021-03-29

**Authors:** Ziyi Wang, Hao Wang, Xuejiao Chen, Sheng Han, Yulin Zhu, Hanhua Wang, Feng Cheng, Liyong Pu

**Affiliations:** ^1^Hepatobiliary Center, The First Affiliated Hospital of Nanjing Medical University, Nanjing, China; ^2^Key Laboratory of Liver Transplantation, Chinese Academy of Medical Sciences, Nanjing, China; ^3^NHC Key Laboratory of Living Donor Liver Transplantation, Nanjing, China; ^4^Department of Cardiothoracic Surgery, Affiliated Yancheng Clinical School of Nanjing Medical University, China; ^5^Department of Radiation Oncology, Affiliated Yancheng Clinical School of Nanjing Medical University, China

## Abstract

At present, liver ischemia-reperfusion (IR) injury is still a great challenge for clinical liver partial resection and liver transplantation. The innate immunity regulated by liver macrophages orchestrates the cascade of IR inflammation and acts as a bridge. As a specific macrophage subunit of vacuolar ATPase, ATP6V0D2 (V-ATPase D2 subunit) has been shown to promote the formation of autophagolysosome in vitro. Our research fills a gap which has existed in the study of inflammatory stress about the V-ATPase subunit ATP6V0D2 in liver macrophages. We first found that the expression of specific ATP6V0D2 in liver macrophages was upregulated with the induction of inflammatory cascade after liver IR surgery, and knockdown of ATP6V0D2 resulted in increased secretion of proinflammatory factors and chemokines, which enhanced activation of NLRP3 and aggravation of liver injury. Further studies found that the exacerbated activation of NLRP3 was related to the autophagic flux regulated by ATP6V0D2. Knocking down ATP6V0D2 impaired the formation of autophagolysosome and aggravated liver IR injury through nonspecific V-ATPase activation independent of V-ATPase-Notchl-Hesl signal axis. In general, we illustrated that the expression of ATP6V0D2 in liver macrophages was upregulated after liver IR, and by gradually promoting the formation of autophagolysosomes to increase autophagy flux to limit the activation of liver inflammation, this regulation is independent of the Notch1-Hes1 signal axis.

## 1. Introduction

Embryonic-derived macrophages act as sentinels of the innate immune response and settle in a sinusoidal arrangement in the liver vasculature. Kupffer cells sense aseptic ischemia-reperfusion injury through TLR and release DAMPs, then extend their “tentacles” to engulf cell debris and remove foreign objects, thereby helping to gradually restore tissue regeneration [1–3]. The cascade of tissue inflammation after ischemia-reperfusion injury is an active process sophisticatedly orchestrated by the synergy between innate immune cells [4, 5]. It is a key step to restore tissue homeostasis to clear infiltrating neutrophils and gradually reprogram monocyte-derived macrophage cells differentiate from proinflammatory to repair phenotype [6–8]. The mixed expression of macrophages of different subgroups after inflammatory activation also provides many challenges for the study of the mechanism of IR damage in different organs [9–11]. F4/80 + CD11b-Ly6C-Kupffer cells can usually limit the excessive inflammation caused by IR to protect the liver [12, 13].

Autophagy degrades impaired proteins and organelles engulfed by autophagosomes through a lysosome-dependent pathway to maintain cell homeostasis. Autophagy participates in the immune regulation of a variety of cells in the immune cascade [14, 15]. IR-induced autophagy activation can clear damaged mitochondria and oxygen free radicals (ROS) and increase energy supply, thereby reducing liver injury [16]. Autophagy has been shown to have multiple mechanisms of crosstalk with the activation of NLRP3 inflammasomes [17–20]. Autophagy clears mitochondria that are damaged during the fight against inflammation, which is the upstream activation signal of NLRP3 inflammasome activation, thereby downregulating the inflammatory response [18]. mtROS and cardiolipin can induce the activation of NLRP3, but it has been found that mitochondrial damage is upstream of caspase1 activation and is independent of the caspase1 activation pathway. The traditional view is that NF-*κ*B promotes the activation of NLRP3 and induces the progression of inflammation, but recent studies suggest that NF-*κ*B can also prevent excessive inflammation by delaying the accumulation of P62 and inhibit the activation of NLRP3 inflammasomes. Macrophage-specific p62 resection leads to significant mitochondrial accumulation and excessive IL-1*β*-dependent inflammation, thereby increasing the death of macrophages [21]. This has triggered our thinking about the inflammatory cascade caused by liver IR that involves inflammation activation and resolution. In addition, when LC3B and Beclin1 are depleted to inhibit the induction of autophagy, NLRP3-related caspase1, IL1*β*, and IL18 will be intensified and activated. Damage to autophagy causes the damaged intracellular mitochondria to not be cleared in time, releases excessive ROS, and also induces NLRP3 activation [19]. Rotenone increases the production of reactive oxygen species in mitochondria and can induce apoptosis. Administration of glutathione and NAC can reverse the induction of apoptosis [7].

Vacuole ATPase (V-ATPases) is an electric proton pump that exists in the intracellular organelles of all eukaryotes. The acidic pH produced by V-ATPase is required for various cellular processes, such as endosomal ligand-receptor dissociation and lysosomal degradation [22, 23]. V-ATPase is composed of two main functional domains, V1 and V0. V1 is mainly responsible for ATP hydrolysis, and V0 is responsible for the transport of protons across the membrane. V-ATPase is the main proton pump for acidifying endosomes and lysosomes. V-ATPase depletion in Atp6v0c mutant mice affects intraluminal acidification and endocytosis [24, 25]. V-ATPases also work through a pathway that does not depend on lysosomal acidification, such as fusion of lysosomes with other organelles to form larger compartments [26, 27]. Here implies the possibility of V-ATPases playing a role in the fusion of autophagosomes and lysosomes. Studies based on HEK293T and BMDM have indeed proved that ATP6V0D2 (V-ATPase D2 subunit) is crucial for the effective formation of phagolysosomes [28]. After severe burns, the activity of myocardial lysosomal V-ATPase decreases in rats, which inhibits myocardial autophagy flux and causes myocardial injury [29]. In the liver, V-ATPase can regulate the cytoplasmic pH of hepatocytes [30]. V-ATPase acidification in rat hepatocytes mediates EGF signal transduction to mTORC1 [31]. The efficiency of liver cancer vaccination significantly improved by targeting the MET-V-ATPase-mTOR axis [32]. Research based on hepatocytes shows that TMEM9-V-ATPase can overactivate Wnt signal through lysosomal degradation of APC, thereby promoting liver regeneration [33]. The inherited liver assembly defects of V-ATPase can cause endoplasmic reticulum stress and liver steatosis [34].

There is also crosstalk between V-ATPases and Notch1 signals. The consumption of TFEB attenuates the ligand-independent Notch signaling by reducing the activity of V-ATPase in human breast epithelial cells [35]. Destroying V-ATPase activity will cause abnormal transport of the *γ*-secretase complex, thereby preventing the cleavage of Notch S3. The lack of a2 subunit will disrupt the lysosomal pathway of Notch and TGF signaling [36, 37]. The most important thing is that the role of Notch signaling in liver cells when facing IR inflammatory activation has been deeply recognized [38, 39].

Therefore, in this study, we highlight the role of ATP6V0D2 in regulating the autophagic flux of macrophages after liver IR surgery, furthermore, detected the mechanism of ATP6V0D2 affecting autophagy degradation and NLRP3 activation. Most importantly, we ruled out the contribution of Notch1 to ATP6V0D2 in regulating liver aseptic inflammation.

## 2. Methods and Materials

### 2.1. Mouse Liver Warm Ischemia Reperfusion Injury Model and Treatment

Using 6-8 weeks of male C57 mice, it was fasted 12 h before surgery, followed by suction anesthesia with 2% isoflurane. Then, cut the abdomen to expose the liver, using an atraumatic clip to interrupt the arterial/portal venous blood supply to the cephalad liver lobes. The left/middle portal vein and the branches of the hepatic artery were clamped with an atraumatic clip to make 70% of the liver ischemic for 90 min. Then, remove the clip to start reperfusion, and the mouse is placed on a constant temperature heating pad to keep warm. Sham controls underwent the same surgery procedure but without vascular occlusion. The mice were sacrificed at different time points after reperfusion.

### 2.2. Isolation and Culture of Liver Macrophages and BMDMs

The liver was perfused in situ with HBSS solution through portal vein fixation, and then, the liver was perfused with type IV collagenase through a constant flow pump. The liver was then removed under aseptic conditions and washed with PBS, then placed in a precooled sterile petri dish; the gallbladder was cut off, the liver tissue was bluntly separated, 2.5 ml of type IV collagenase was added to the petri dish, and the mixture was mixed. Place it on a 37°C water bath shaker and shake in the water bath and fully digest. The suspension is transferred to a 70 *μ*m cell filtrator and filtered to a petri dish, and the filtered suspension is the liver single cell suspension. After centrifugation, add 50% Percoll solution, 25% Percoll solution, and liver single cell suspension in sequence for 15 minutes and then aspirate the 50% Percoll solution and 25% Percoll solution. The middle cell layer is centrifuged, and the cell pellet is cultured at 37°C and 5% CO2. Continue to culture in the box for 2 hours and then take it out to remove nonadherent cells to obtain adherent macrophages.

Bone marrow cells were isolated from mouse femurs and tibias. Cells were cultured in DMEM supplemented with 10% FBS and 20% L929-conditioned medium for 1 week. Bone marrow-derived macrophages (BMDMs) were then replated and cultured overnight in new culture dishes for further experiments.

### 2.3. Liver Histopathology and Immunohistochemistry

Collect liver tissues and soak them in 4% paraformaldehyde for at least 24 hours and then slice the tissues after embedding in paraffin. Sections (4 *μ*m) were stained with H&E, and inflammation and tissue damage were observed through an optical microscope. The degree of liver IRI tissue damage was blindly evaluated from 0 to 4 points by the standard scores established by Suzuki.

### 2.4. Western Blot

The liver tissues or cell lysates of the operation group and the control group were extracted with cell lysis buffer (1% Triton X-100, 0.5% sodium deoxycholate, 0.1% SDS, 10% glycerol, 20 mM Tris (pH 7.4), 137 mM Nacl). The protein was subjected to 10% or 12% SDS-PAGE electrophoresis and then transferred to PVDF nitrocellulose membrane. Antibodies against ATP6V0D2 (Sigma-Aldrich), Hes1 (Cell Signaling Technology), NLRP3, IL-1*β*, ASC, LC3B, SQSTM1, and *β*-actin (Abcam) were used for detection.

### 2.5. qRT-PCR

Total mRNA was extracted from frozen cells and tissues using Trizol (Invitrogen, California, USA) and reverse transcribed into cDNA using Transcriptor First Strand cDNA Synthesis Kit (Roche, Indiana, USA). The samples were then subjected to real-time quantitative qPCR measurement (Roche, Indiana, USA) with SYBR Green fluorescent dye. The expression level and results of the target gene are standardized for the GAPDH expression.

### 2.6. Plasmid Construction and Lentiviral Vector Production

Designed a double-stranded siRNA targeting the target sequence and the control siRNA (siControl) chemically synthesized by Sangon Biotech (Shanghai, China), then were proven to have specificity through NCBI BLAST. Construct a BR-V lentiviral vector (BR-V lentiviral vector, pMD2.G vector, and pSPAX2 vector, YBR Biotech, Shanghai) consisting of ATP6V0D2 and Notch1 siRNA plasmids.

### 2.7. ELISA Measurement

Measure TNF-*α*, IL-1*β*, IL-6, IL-10, IL-18 (R&D Biosystem), and sALT and sAST (MlBio, Shanghai) in mouse Kupffer cell culture supernatant or mouse serum according to the manufacturer's kit instructions.

### 2.8. In Vivo siRNA Knockdown

Mix ATP6V0D2, Notch1, and BECN1 siRNA (Sangon Biotech, Shanghai, China) with mannose-conjugated polymer (polyplus-transfection SA, Illkirch, France) according to the manufacturer's instructions, injected through tail vein 4 h before performing IR surgery on mice (siRNA 2 mg/kg) to achieve knockdown in liver macrophages.

### 2.9. Transmission Electron Microscopy and Confocal Immunofluorescence

#### 2.9.1. Transmission Electron Microscopy

Fixing solution for electron microscope (ServiceBio, Wuhan China) fixed at 4°C and centrifugation at low speed, 1% agarose coating, 0.1 M PBS (pH 7.4) rinsing, and 1% osmic acid·0.1 M phosphate buffer PB (pH 7. 4). After fixing at room temperature (20°C), rinse with 0.1 M phosphate buffer PB (pH 7.4) and then dehydrated with alcohol-acetone. Acetone and 812 embedding agent (SPI) are infiltrated overnight in the same proportion. After pure 812 is embedded, the sample is inserted into the embedding plate and then 37°C oven overnight. The sample is then placed in a 60°C oven to polymerize for 48 h. After embedding, use an ultrathin microtome (Leica UC7) and double stain with uranium and lead (2% uranyl acetate saturated alcohol solution, lead citrate). After drying, observe through transmission electron microscope (HITACHI HT7700), collect images, and analyze.

#### 2.9.2. Confocal Immunofluorescence Microscope

Cover the sample with 3% BSA uniformly. After blocking at room temperature for 30 minutes, discard the blocking solution. Add the prepared primary antibody dropwise to the cell plate. Place the cell culture plate flat in a humid box and incubate overnight at 4°C. On the next day, the cell plate was placed on a decolorizing shaker and washed 3 times with shaking for 5 minutes each time. After a little spin-drying, drop the samples in the histochemistry kit with the secondary antibody corresponding to the primary antibody to cover the sample and incubate at room temperature for 50 minutes. Then, take out the climbing piece and place it in PBS (pH 7.4) on a decolorizing shaker, shaking and washing 3 times, 5 minutes each time. After the sections were shaken dry, DAPI dye solution was added dropwise to the circle and incubated for 10 minutes at room temperature in the dark. The slides were placed in PBS (pH 7.4) and washed 3 times with shaking on a decolorizing shaker, each time for 5 minutes. The slides were dried slightly and then mounted with antifluorescence quenching mounting tablets. The slices were observed under a confocal fluorescence microscope (NIKON ECLIPSE C1), and images were collected.

### 2.10. Statistical Analysis

The results are shown as mean ± SD. Use one-way ANOVA and Student *t*-test to analyze and compare the data. All data analysis was done using ImageJ and GraphPad Prism 8.0. The *P* value is considered statistically significant in the case of two-tailed and < 0.05.

## 3. Results

### 3.1. Liver Ischemia-Reperfusion Specifically Regulates the Expression of ATP6V0D2 in Macrophages

**Figure 1:**
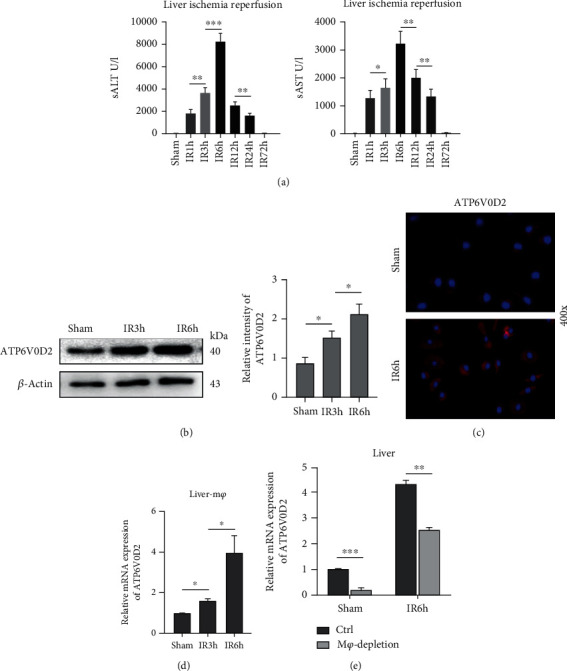
The expression of ATP6V0D2 in liver macrophages is upregulated by IR. 90 min liver warm ischemia reperfusion model was constructed as described in the Methods and Materials. (a) Serum alanine aminotransferase (sALT) and aspartate aminotransferase (sAST) (*n* = 4‐6 mice/group) were used to evaluate the liver inflammatory injury at 1, 3, 6, 12, 24 h, and 72 h after IR, sham as control. (b) Protein level of liver ATP6V0D2 was detected by western blot at 3 h and 6 h after IR, sham as control. (c) The expression of ATP6V0D2 in liver macrophages at 6 h after IR was detected by immunofluorescence staining, sham as control (400x magnification; representative of three experiments). (d) The expression of ATP6V0D2 in liver M*φ* at 3 h and 6 h after IR was measured by quantitative real-time-PCR (*n* = 3‐6 mice/group), sham as control. (e) Clodronate liposome was pretreated (i.v, 250 *μ*l/mouse) to eliminate liver M*φ* 24 h before IR surgery, then detect the expression of ATP6V0D2 in liver by qRT-PCR at 6 h after IR (*n* = 3‐6 mice/group), sham as control. ^∗^*P* < 0.05. ^∗∗^*P* < 0.01. ^∗∗∗^*P* < 0.005.

Unexpectedly, the expression of ATP6V0D2 was most severely inhibited in LPS-induced inflammatory models in vitro; this could be understood as LPS limits the ability of engulfing microbes and clear inflammation and inhibits transcription [28]. Therefore, it is urgent to know the expression of liver ATP6V0D2 in the context of different mechanisms of activating injury and organ specificity, as well as whether it is related to the specificity of liver macrophages. We have done some work in advance to prove that the liver inflammation reaches its peak at 6 hours after 90 minutes of warm ischemia-reperfusion in mice (Figure 1(a)). Therefore, we examined the expression of ATP6V0D2 in the sham group, IR3h, and IR6h. In contrast to LPS induction, the intense inflammatory response induced by IR activates the expression of ATP6V0D2 in the liver. An analysis of multiple organs showed that the expression of ATP6V0D2 was not high in quiescent liver [40]. This is also consistent with our findings. However, the expression of ATP6V0D2 was upregulated in response to the inflammatory activation of IR, which may be partly due to the differentiation and heterogeneity of liver macrophages, namely, the fact that ATP6V0D2 plays a key role in monocyte-derived macrophages (Figures 1(b)–1(d)), therefore requires our further verification. We applied Clodronate liposome to preclear resident macrophages of the liver and in deed basically eliminated the expression of ATP6V0D2 mRNA in the sham group. However, in the IR6h group, the expression of ATP6V0D2 in the liver is still relatively high even after the preclearance of resident macrophages (Figure 1(e)). On the one hand, we demonstrated the specificity of the expression of ATP6V0D2 in mouse liver, and on the other hand, we preliminarily described the picture showing how V-ATPase subunit-ATP6V0D2 in different subsets of macrophages regulates liver inflammation, although few relevant research has been reported before.

### 3.2. siATP6V0D2 Leads to Aggravated IR Inflammation and Tissue Injury

**Figure 2:**
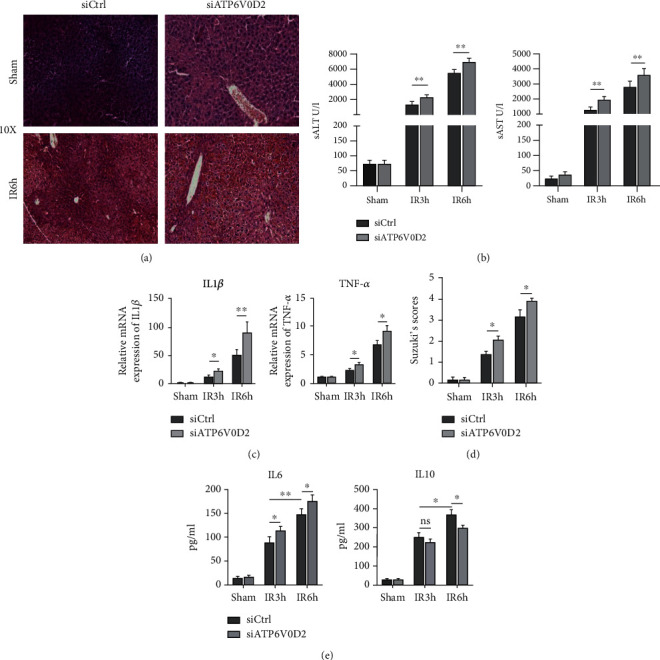
Knocking down ATP6V0D2 aggravates IR inflammation and tissue damage. (a) H&E staining was used to evaluate the liver injury of siATP6V0D2 pretreated groups and siCtrl groups at 6 h after IR, sham as control. (b) sALT and sAST (*n* = 4‐6 mice/group) were used to evaluate the liver injury at 3 and 6 h after IR in siATP6V0D2 group, siCtrl as control. ^∗∗^*P* < 0.01. (c) The expression of chemokines responsible for the inflammatory M*φ*s at 3 h and 6 h after IR in siATP6V0D2 and siCtrl groups was measured by quantitative real-time-PCR (*n* = 3‐6 mice/group), sham as control. (d) Suzuki scores were used to evaluate liver injury (*n* = 3‐6 mice/group). (e) Liver macrophages were transfected with siATP6V0D2 or siCtrl for 4 h and then treated with IR surgery. Serum IL-6 and IL-10 were measured by ELISA at 3 h or 6 h after IR (*n* = 4‐6 mice/group).

ATP6V0D2 is undoubtedly involved in the regulation of the inflammation of liver IR. To achieve the purpose of research in vivo, a relatively specific mannose conjugated vector (polyplus-transfection SA, Illkirch, France) anchored to liver macrophages was used as vector to deliver ATP6V0D2 siRNA plasmid (Sangon Biotech, Shanghai). Compared with the sham group, preknockdown of ATP6V0D2 caused further aggravation of liver tissue injury at different time points after IR surgery and correspondingly delayed tissue injury repair. Evaluation based on Suzuki's scores also demonstrated that siATP6V0D2 caused increased liver injury (Figures 2(a) and 2(d)). Correspondingly, before reaching the peak of inflammation after IR, siATP6V0D2 will aggravate the induction of inflammation, increase the content of proinflammatory factors, and decrease the content of anti-inflammatory factors in serum (Figures 2(b)–2(e)). In general, siATP6V0D2 impaired the ability of macrophages to protect the liver and to clear inflammation, thus leading to increased activation of IR inflammation and aggravating postoperative liver tissue damage.

### 3.3. siATP6V0D2 in Liver Macrophages Exacerbates the Activation of NLRP3 Induced by IR

**Figure 3:**
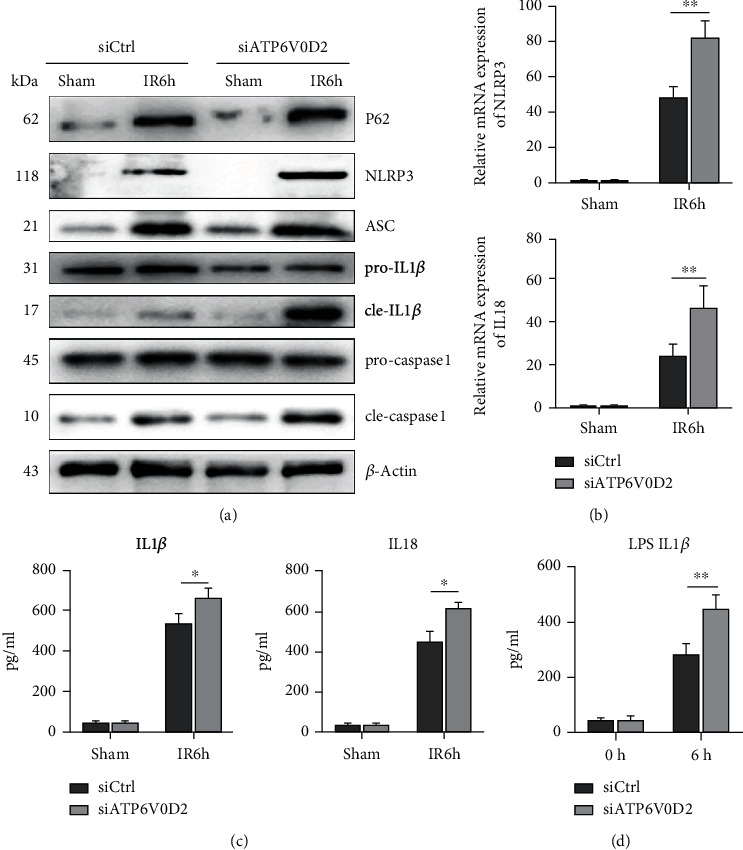
Knockdown of ATP6V0D2 increased the activation of NLRP3 in liver IR macrophages. (a) The protein levels of P62, NLRP3, cleaved-caspase1, pro-caspase1, ASC, cleaved-IL1*β*, pro-IL1*β*, and *β*-actin in siATP6V0D2 and siCtrl groups were harvested at IR 6 h, then detected by western blot (representative of 3-6 experiments). (b) The expressions of NLRP3 and IL18 in macrophages harvested from siATP6V0D2 and siCtrl IR6h groups were measured by quantitative real-time-PCR (*n* = 4‐6 mice/group). ^∗∗^*P* < 0.01. (c) Isolated macrophages from different experimental groups were cultured for another 6 h in vitro. IL-1*β* and IL-18 levels were measured in the culture supernatant by ELISA (*n* = 4‐6 mice/group). (d) Liver macrophages were transfected with siATP6V0D2 or siCtrl for 24 h and then stimulated with LPS for 6 h in vitro; then, the expression of IL1*β* was measured in the culture supernatant by ELISA (*n* = 4‐6 mice/group).

It has been proved that knockdown of ATP6V0D2 leads to enhanced inflammasome activation in BMDMs and in LPS-induced peritonitis [28]. Therefore, in order to explore the role of ATP6V0D2 in secondary inflammation of liver IR, warm ischemia reperfusion surgery was performed on C57 mice for 90 min after knocking down ATP6V0D2 in vivo. Preknocking down ATP6V0D2 increases P62 accumulation after IR surgery, enhancing the induction of NLRP3, which upregulated expressions of NLRP3, ASC, cleaved caspase-1, and cleaved IL-1*β* are relatively upregulated (Figures 3(a)–3(c)). In vitro, compared with wild type, liver macrophages which knocked down ATP6V0D2 stimulated with LPS resulted in increased secretion of IL1*β* (Figure 3(d)).

### 3.4. Knockdown of ATP6V0D2 Aggravates ROS-Related Mitochondrial Damage after IR

It is well known that production of ROS caused by mitochondrial damage is one of the main inducers of NLRP3 activation. Therefore, we want to know whether the enhanced activation of NLRP3 caused by siATP6V0D2 was related to the aggravation of mitochondrial damage in the macrophages after IR. After knocking down ATP6V0D2 in vivo in advance, we observed the changes of mitochondria during the inflammatory activation induced by IR surgery by TEM. IR surgery without intervention causes mitochondrial damage in the form of enlarged mitochondria, increased electron density, and partially dissolved intramembrane matrix.

**Figure 4:**
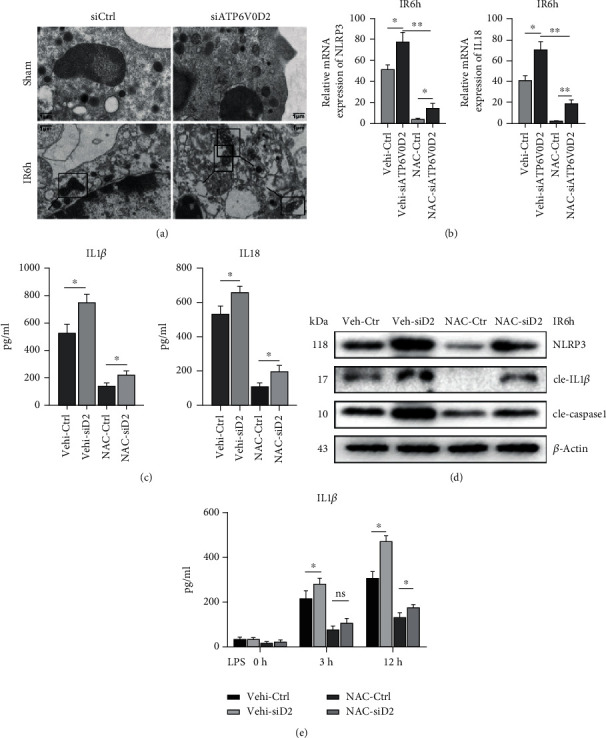
Knockdown of ATP6V0D2 aggravates ROS-related mitochondrial damage after IR. (a) Mitochondria in liver macrophages were detected by transmission electron microscopy at IR6h; the areas marked by box were mitochondria (1000x magnification; scale bars, 1 *μ*m; representative of three experiments). (b) NAC was pretreated (150 mg/kg, ip) 30 min before IR surgery, then detected the expression of NLRP3 and IL18 in macrophages harvested from siATP6V0D2 and siCtrl IR6h groups by quantitative real-time-PCR (*n* = 4‐6 mice/group). ^∗∗^*P* < 0.01. (c) After pretreating NAC or vehicle, isolated macrophages from siATP6V0D2 and siCtrl groups were cultured for another 6 h in vitro. IL-1*β* and IL-18 levels were measured in the culture supernatant by ELISA (*n* = 4‐6 mice/group). (d) On the basis of pretreating NAC or vehicle, the levels represented for activation of NLRP3-related proteins were detected by western blot in siATP6V0D2 and siCtrl groups at IR6h (representative of three experiments). (e) BMDMs were transfected with siATP6V0D2 or siCtrl for 24 h and then stimulated with LPS for 0, 3, and 6 h in vitro. NAC (5 mM) or ctrl was treated 1 h before LPS stimulation. IL-1*β* in the culture supernatant was measured by ELISA (*n* = 4‐6 mice/group).

Unexpectedly, knockdown of ATP6V0D2 can cause further enlargement and vacuolation of mitochondria after IR, as well as the phenomenon of ridge fracture, disappearance, and membrane rupture, indicating severe damage of mitochondria (Figure 4(a)). Our experiments in vivo demonstrated that further knockdown of ATP6V0D2 inhibited the induction of NLRP3 and IL-1*β* and decreased secretion of active IL-1*β* and IL-18, compared with the vehicle group, when ROS were cleared by NAC in advance (Figures 4(b) and 4(c)). These results illustrate the negative correlation between ATP6V0D2 expression and ROS activation. It is worth noting that although depleting ROS largely inhibited the activation of inflammasomes, it does not completely prevent it (Figure 4(d)). Then, we treated BMDMs which transfected with siATP6V0D2 and siCtrl with LPS and examined the secretion of IL-1*β* in the culture supernatant at 3 h and 12 h after stimulation. We found that the preuse of NAC to clear intracellular ROS resulted in reduced secretion of IL-1*β*, while siATP6V0D2 reversed the reduction effect. (Figure 4(e)).

### 3.5. siATP6V0D2 Regulates Activation of NLRP3 by Impairing the Autophagic Flux after IR Surgery

**Figure 5:**
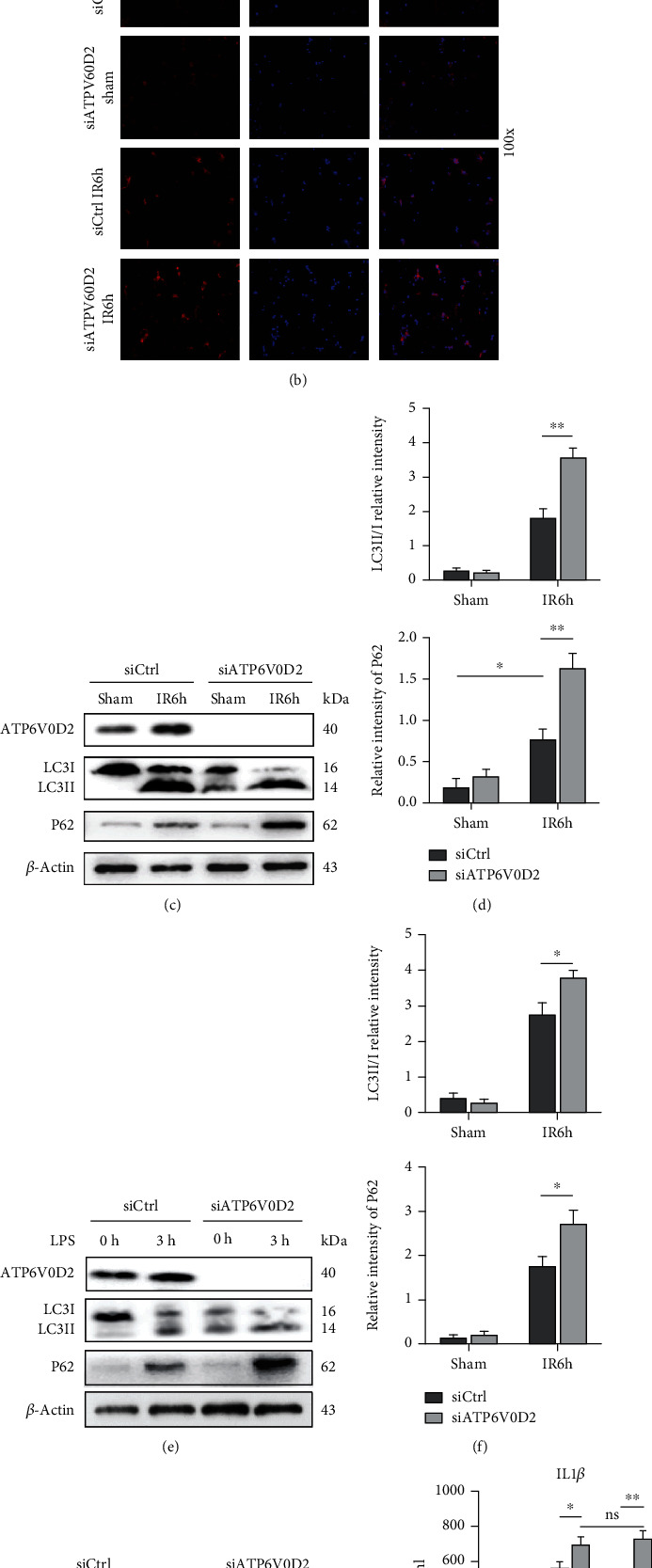
siATP6V0D2 induces NLRP3 activation by impairing IR autophagy flux. (a) The detection of autophagic microstructures in liver macrophages by transmission electron microscopy at IR 6 h; the areas marked by box were autophagolysosome, solid triangle arrows were lysosomes, and hollow triangle arrows were autophagosomes (1000x magnification; scale bars,1 *μ*m; representative of three experiments). (b) Immunofluorescence staining of LC3B in macrophages from siATP6V0D2 and siCtrl groups was detected by confocal microscopy at IR 6 h, sham as control (100x magnification; representative of three experiments). (c) The protein levels of LC3B and P62 in M*φ*s from siATP6V0D2 and siCtrl groups were detected by western blot at 6 h after IR (representative of three experiments). (d) LC3II/I relative intensity and P62/GAPDH relative intensity were analyzed by Prism. (e) BMDMs were transfected with siATP6V0D2 or siCtrl for 24 h and then stimulated with LPS for 0 h and 3 h in vitro. The protein levels of LC3B and P62 in M*φ*s were detected by western blot (representative of three experiments). (f) LC3II/I relative intensity and P62/GAPDH relative intensity of macrophages stimulated with LPS were analyzed by Prism. (g, h) Macrophages at IR6h which pretreated with siATP6V0D2 or siCtrl for 4 h or additional rapamycin (5 mg/kg, ip) for 1 h were harvested in vitro. The protein levels of LC3B and P62 in M*φ*s were detected by western blot (representative of three experiments).

Blocking autophagy leads to the defective clearance of damaged mitochondria, which results in the accumulation of ROS and further leads to the activation of NLRP3. Roughly speaking, the threshold of mitochondrial dysfunction will trigger the induction of NLRP3 [18]. We observed the morphological changes of macrophages after IR operation under transmission electron microscope and found that compared with the control group, the knockdown of ATP6V0D2 would lead to the reduced formation of autophagolysosomes. More primary lysosomes and atypical autophagosomes were scattered in the cytoplasm of macrophages that knocked down ATP6V0D2, and they showed a tendency to combine with each other (Figure 5(a)). The results of immunofluorescence suggest that knocking down ATP6V0D2 will cause more LC3B marked with red fluorescence in macrophages to show clumps or crescent-shaped accumulations, suggesting induction of autophagy or inhibition of autophagy degradation (Figure 5(b)). On this basis, we carried out a western blot to research and found that preknockdown of ATP6V0D2 in vivo caused accumulation of LC3II and P62 (Figures 5(c)–5(f)). We previously demonstrated that knockdown ATP6V0D2 leads to the induction of NLRP3, and we wondered if this was related to ATP6V0D2's regulation of autophagy. Rapamycin, an mTOR inhibitor, can induce autophagy.

Rapamycin (5 mg/kg) was injected through tail vein to induce autophagy before establishing IR models in mice. In the control group, mice were treated with Rapa in advance to induce IR autophagy, leading to degradation of P62 and inhibition of NLRP3 activation. However, when the expression of ATP6V0D2 was suppressed in advance, although Rapamycin was still used to induce autophagy, the degradation of P62 and the inhibitory effect of NLRP3 were both impaired. It shows that ATP6V0D2 plays a regulatory role in autophagy independent of the period when autophagy is induced, and it mainly takes part in the degradation process of autophagolysosomes. The activation of NLRP3 induced by knocking down ATP6V0D2 is also dependent on its inhibitory effect on autophagy flux (Figure 5(g)). ELISA determination of macrophages cultured in vitro after surgery also proved the inherent correlation of ATP6V0D2's inhibiting NLRP3 activation by promoting autophagy (Figure 5(h)).

### 3.6. siATP6V0D2 Impaires the Formation of Autophagolysosome after IR

**Figure 6:**
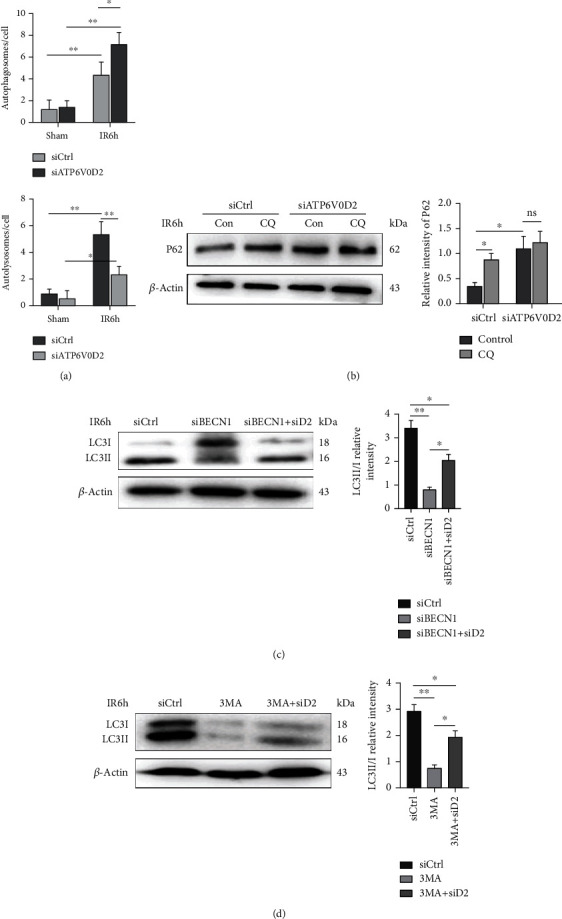
siATP6V0D2 impairs autophagolysosome formation after IR. (a) The average number of autophagosomes and autolysosomes at IR6h in each macrophage harvested from siATP6V0D2 and siCtrl groups. (b) Macrophages at 6 h after IR which pretreated with siATP6V0D2 or siCtrl were harvested and treated with CQ (20 nM) for another 4 h in vitro. The protein levels of P62 were detected by western blot (representative of three experiments). (c) The protein levels of LC3B and *β*-actin in siBECN1 or siBECN1 + ATP6V0D2 (200 *μ*l, iv)or siCtrl groups were harvested at IR 6 h, then detected by western blot (representative of 3-6 experiments). (d) The protein levels of LC3B and *β*-actin in 3MA or 3MA + ATP6V0D2 (200 *μ*l, iv) or siCtrl groups were harvested at IR 6 h, then detected by western blot (representative of 4-6 experiments).

Transmission electron microscopy was used to observe the formation of autophagosomes and autophagolysosomes in hepatic macrophages in the control group and the ATP6V0D2 knockdown group. Not surprisingly, the knockdown of ATP6V0D2 caused more autophagosomes to be redundant and fewer autophagolysosomes to be formed. At the same time, a large number of primary lysosomes and secondary lysosomes were found in the operation groups in which ATP6V0D2 was knocked down (Figure 6(a)). In order to further prove that it is during the period of autophagy degradation that ATP6V0D2 plays a role, we injected siATP6V0D2 and siBECN1 in vivo as described above for verification, and 3MA (30 mg/kg) was injected through abdomen. CQ is a commonly inhibitor of autophagosome and lysosome fusion. The use of siATP6V0D2 or CQ alone would lead to the accumulation of P62, but the expression of P62 was not further upregulated when both of them were used together (Figure 6(b)). Beclin1 is known to play an important role in the formation of autophagosomes. siBECN1 significantly inhibited the lipidation of LC3B, and the lipidation of LC3B was reversed by using siATP6V0D2 (Figure 6(c)). This can be understood that the degradation of LC3B was impaired in the autophagosome which formed finitely. When we combined the autophagy inhibitor 3MA and siATP6V0D2, we found a similar effect to siBECN1 (Figure 6(d)). Our results indicate that ATP6V0D2 plays a key role in impairing the formation of autophagolysosomes during autophagy degradation period.

### 3.7. ATP6V0D2 Is Independent of V-ATPase-Notch1 to Reduce IR Injury

There has been a great deal of evidence that Notch signal transduction requires the activity of V-ATPase. V-ATPase acts on the upstream of the *γ*-secretase complex and is essential for the release of the Notch intracellular domain (NICD) [35, 41, 42].

**Figure 7:**
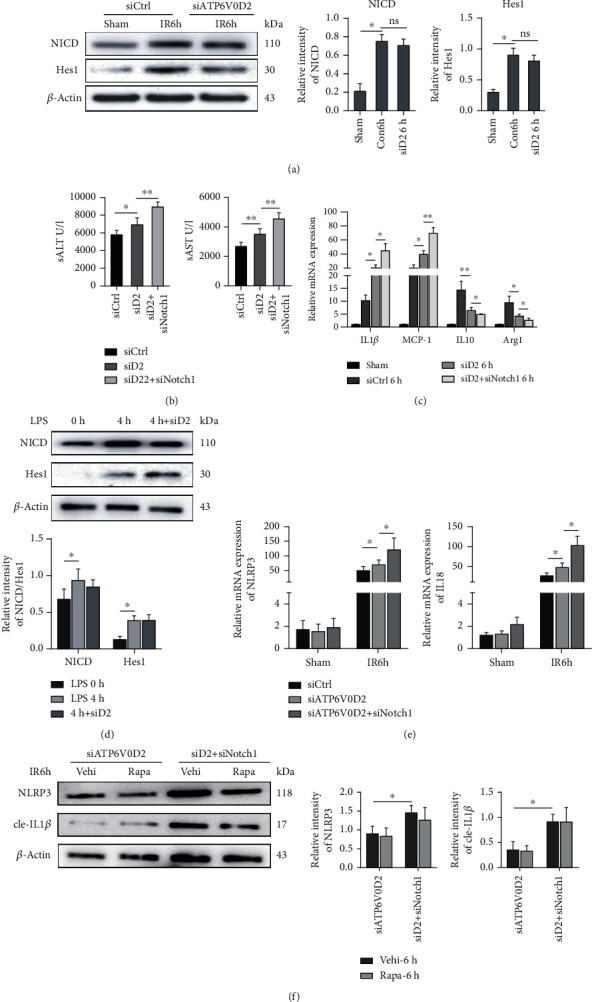
The function of ATP6V0D2 is independent of the V-ATPase activity that induces Notch1 signaling. (a) Mannose-siATP6V0D2 conjugated vector was injected into the tail vein before IR operation, and then, the protein levels of NICD and Hes1 of macrophages were detected at 6 h after IR by western blot.(b) Injection of siATP6V0D2 (iv) or siNotch1 + siATP6V0D2 4 h before IR surgery, siCtrl as control. Then, detected the level of ALT and AST in serum at IR 6 h by ELISA. (c) Relative expression of IL1*β*, MCP-1, IL10, and Arg1 in liver macrophages quantitied by qPCR. (d) BMDMs were transfected with siATP6V0D2 or siCtrl for 24 h, then stimulated with LPS for 0 h or 4 h, harvested the cells to detect the protein levels of NICD and Hes1 by western blot. (e) Injection of siATP6V0D2 (iv) or siNotch1 + siATP6V0D2 4 h before IR surgery, siCtrl as control. Then, detected the mRNA levels of NLRP3 and IL18 at 6 h by RT-qPCR (representative of 4-6 experiments). (f) Macrophages at IR6h which pretreated with siATP6V0D2 or siATP6V0D2 + siNotch1 for 4 h or additional rapamycin (5 mg/kg, ip) for 1 h were harvested in vitro. The protein levels of NLRP3 and cle-IL1*β* in M*φ*s were detected by western blot (representative of three experiments).

Therefore, we want to explore whether ATP6V0D2 reduces inflammation after liver IR surgery due to activation of the Notch signaling cascade in macrophages. We injected the siATP6V0D2 plasmid with mannose-conjugated vectors into the tail vein before IR surgery and then detected the expression of NICD and Hes1 at 6 h. siATP6V0D2 did not cause significant changes in the expression of NICD and Hes1 (Figure 7(a)). Simultaneous injection of siATP6V0D2 and siNotch1 (40 *μ*g/mouse, repeated twice) together or siATP6V0D2 alone into the tail vein 4 hours before IR surgery and then detect the liver injury after the pretreatment. On the basis of knocking down ATP6V0D2, further knocking down Notch1 caused a significant increase in liver inflammatory damage, proving that Notch1 is not downstream of ATP6V0D2 (Figures 7(b) and 7(c)). We verified our hypothesis on BMDMs in vitro and got similar conclusions (Figure 7(d)). Further, we explored the relationship between ATP6V0D2 and Notch1 in autophagy and NLRP3 activation. We found that compared to knocking down ATP6V0D2, further knocking down siNotch1 caused significant transcriptional activation of NLRP3 and IL18. This shows that Notch1 is not downstream of ATPV0D2 (Figure 7(e)). Then, we further studied the roles of the two in autophagy induction. As previously shown, preinduction of autophagy did not reduce the activation of NLRP3 in the siATP6V0D2 group, while NLRP3 and cle-IL1*β* in the siATP6V0D2 + siNotch1 group had higher protein levels than the siATP6V0D2 group. The preinduction of autophagy did not have a significant effect on the results (Figure 7(f)).

## 4. Discussion

Ischemia-reperfusion injury is one of the bottlenecks that limit the development of liver surgery. Aseptic injury induces liver macrophages to participate in the innate immune response. Different subsets of liver macrophages are programmed in a cascade to jointly orchestrate the inflammatory response of the liver. Autophagy is involved in the regulation of inflammation after liver IR. Pretreatment of isoflurane activates AMPK signals in hepatocytes, which in turn induces autophagy and reduces liver IR injury [43]. Starvation induces autophagy in macrophages through Sirt1, thereby reducing the level of HMGB1 and ultimately protecting the liver from IR injury [44].

Vacuole ATPase (V-ATPases) is a large protein complex, essentially an electric proton pump, composed of the outer V1 domain that hydrolyzes ATP and the complete V0 domain that transports hydrogen ions, ensuring the acidification of the endolysosome compartment, which is the basic process of cargo transportation, classification, and degradation during endocytosis and autophagy. The acidified environment is also critical for protein degradation and the recovery of amino acid transporters coupled with protons [22, 23, 45]. The earliest research of cells lacking V-ATPase function in Drosophila showed impaired acidification of the body compartment of the lysosome and the related failure to degrade endocytic cargo [25]. V-ATPase subunits in the V1 and V0 domains, including the D subunit, play a coordinated role to control the coupling of proton transport and ATP hydrolysis [40]. However, previous studies have shown that the D1 subunit synergistically responsible for promoting the acidification of macrophage lysosomes and the D2 for promoting macrophage autophagosome-lysosome fusion in vitro [28]. Lactic acid induces the activation of mTORC1 in macrophages, thereby inhibiting the expression of ATP6V0D2 in tumor-associated macrophages and promoting HIF-2*α*-mediated tumor progression [46].

In the liver, pre-IR ischemic preconditioning has a protective effect on Na(+) overload and hepatocyte killing. Blocking V-ATPase activity will impair this effect [47]. Bafilomycin A1 acts as a V-ATPase inhibitor to inhibit the fusion of autophagosomes and lysosomes in liver cancer cells [48]. V-ATPase acidification in rat hepatocytes mediates EGF-mTORC1 signaling to play a regulatory role [31]. TMEM9-V-ATPase can overactivate Wnt signaling through lysosomal degradation of APC in hepatocytes, thereby promoting liver regeneration [33]. The inherited liver assembly defects of V-ATPase can cause endoplasmic reticulum stress and liver steatosis [34].

V-ATPase promotes the degradation of Notch in the lysosome and the activation of Notch signal in the endosome through early endosome acidification [41]. Insufficient Notch1 haploid can increase macrophage M2 polarization [49]. It has been proven that the activity of V-ATPase is essential for the activation of Notch signaling in Drosophila neural stem cells [50]. In human breast epithelial cells, obstruction of the V-ATPase C subunit changes the size and function of the lysosomal compartment, while the consumption of TFEB reduces the nonligand dependence by reducing the activity of V-ATPase dependent Notch signaling [35]. Ligand-dependent Notch signal activated intracellular domain (NICD) is released by proteolytic cleavage by the *γ*-secretase complex, transferred to the nucleus, and then activates the Notch1 target gene Hes1. Lgl promotes the binding of Vap33 and V-ATPase, thereby inhibiting V-ATPase-mediated acidification of endosomal vesicles, reducing *γ*-secretase activity, and inhibiting Notch signal transduction [51]. The destruction of V-ATPase activity can cause abnormal transport of the *γ*-secretase complex, thereby preventing the cleavage of Notch's S3. A2V-ATPase (a2V) can regulate the processing of Notch receptors and change the occurrence of Notch signaling in breast cancer. At the same time, a2V deficiency will destroy the lysosomal pathway in Notch and TGF signaling, thereby impairing breast development.

Notch signal has been proved to be important and complicated in the regulation of liver inflammation stress. The Notch-RBP-J pathway can inhibit the generation of ROS through JAK2/STAT3 signaling, thereby protecting hepatocytes from I/R injury [52]. Myeloid Notch1 deficiency activates the RhoA/ROCK pathway and aggravates hepatocellular damage in mouse ischemic livers [39]. Serelaxin enhances the expression of anti-inflammatory genes by increasing the macrophage Notch1 intracellular domain (NICD) and split-1 (Hes1) to play a hepatoprotective effect in human and murine liver IRI and OLT [38].

Studies have shown that the acidification of lysosomes in macrophages does not depend on ATP6V0D2 but ATP6V0D1 [28]. In addition, lack of ATP6V0D2 does not affect osteoclast differentiation or V-ATPase activity [53]. The crosstalk between V-ATPase and Notch signal mainly depends on the acidification of lysosomes. The low pH environment caused by acidification helps *γ*-secretase activity [35, 37, 45]. Although some researchers have proposed that lactic acid in TAM can activate mTORC1 signal and thereby inhibit TFEB-mediated ATP6V0D2 expression, our data show that ATP6V0D2 reduces liver IR injury mainly by inducing autophagolysosome formation, without significant activation of Notch signal. Nevertheless, due to technical limitations, it is questionable whether we knock down ATP6V0D2 to impair the functions of other subunits of V-ATPase.

In addition, we did not clearly distinguish the heterogeneity of liver macrophages in our experiments. Although there is early research, evidence based on THP-1 cells that the differentiation of cells from monocytes to macrophages leads to a large expansion of lysosomal compartments and an increase in V-ATPase expression [54].

In the future, it is necessary to specifically divide different subsets of macrophages and then to further study the role played by ATP6V0D2. Regardless, our work complements the mapping of the liver IR inflammatory activation mechanism, helping to further understand the role of ATP6V0D2 in the context of liver IR-induced innate immunity. Nevertheless, more detailed research is needed to further explain and dig out our findings.

## Data Availability

All experimental data has been displayed in the manuscript. If necessary, additional raw data including macrophage immunofluorescence, liver staining, transmission electron microscopy, and molecular biology experiments can be obtained from the corresponding authors.

## Supplementary Materials

Supplementary Materials The sequences of qPCR primers referred in the article.
