# Supplementary material for: Inhibiting ATP6V0D2 Aggravates Liver Ischemia-Reperfusion Injury by Promoting NLRP3 Activation via Impairing Autophagic Flux Independent of Notch1/Hes1
Source: J Immunol Res. 2021 Mar 29;2021:6670495. doi: 10.1155/2021/6670495 (PMC8024071; doi:10.1155/2021/6670495)
Supplement: Supplementary Materials — The sequences of qPCR primers referred in the article. [file 6670495.f1.pdf]

Supplementary Table 1: Primer sequences used for Real-Time Quantitative PCR

Gene	Forward	Reverse
NLRP3	5’-CTGGCTGCGGATGGAATTTG-3’	5’-CTGGTCCTTTCCTCACGGTC-3’
IL1 β	5’-TGTAATGAAAGACGGCACACC-3’	5’-TCTTCTTTGGGTATTGCTTGG-3’
IL10	5’-CGACTCCTTAATGCAGGACT-3’	5’-TTGATTTCTGGGCCATGC-3’
Arg1	5'-GGAAAGCCAATGAAGAGCTG-3'	5'-GATGCTTCCAACTGCCAGAC-3'
MCP1	5’-CATCCACGTGTTGGCTCA-3’	5’-GATCATCTTGCTGGTGAATGAGT-3’
ATP6V0D2	5'-TGCGGCAGGCTCTATCCAGAGG-3′	5′-CCACTGCCACCGACAGCGTC-3′
TNF-α	5'-GCCTCTTCTCATTCCTGCTTGT-3'	5’-GATGATCTGAGTGTGAGGGTCTG-3’
